# Changes in Polyphenolic Concentrations of Table Olives (cv. Itrana) Produced Under Different Irrigation Regimes During Spontaneous or Inoculated Fermentation

**DOI:** 10.3389/fmicb.2018.01287

**Published:** 2018-06-15

**Authors:** Giorgia Perpetuini, Giovanni Caruso, Stefania Urbani, Maria Schirone, Sonia Esposto, Aurora Ciarrocchi, Roberta Prete, Natalia Garcia-Gonzalez, Noemi Battistelli, Riccardo Gucci, Maurizio Servili, Rosanna Tofalo, Aldo Corsetti

**Affiliations:** ^1^Faculty of Bioscience and Technology for Food, Agriculture and Environment, University of Teramo, Teramo, Italy; ^2^Department of Agriculture Food and Environment, University of Pisa, Pisa, Italy; ^3^Department of Agricultural Food and Environmental Sciences-DSA3, University of Perugia, Perugia, Italy

**Keywords:** table olive, irrigation, starter cultures, phenolic compounds, oleuropein, Itrana

## Abstract

Irrigation is widely used for the production of table olives because it increases fruit size and yield. However, irrigation also determines less accumulation of total phenols, an increase in water content, a decrease of firmness, lower concentrations of soluble sugars in the mesocarp, thus positively or negatively affecting the fermentation process for the production of table olives. In this study we tested the hypothesis that green fruits of cultivar Itrana obtained by different irrigation regimes had different phenolic concentration that responded differentially to spontaneous or inoculated fermentation. Fruits were harvested from two orchards in the Latina province of Latium, Italy, which had been irrigated with different volumes of water during the growing season to compare the evolution of spontaneous and inoculated fermentation processes. We measured fruit characteristics at harvest, changes in the concentrations of secoiridoids and lignans, and main microbial groups abundance during fermentation. At harvest and during fermentation the concentration of phenolic compounds was higher in fruits sampled from trees that had received less water in the field. Differences were observed between spontaneous and inoculated fermentations, with a prevalence of lactic acid bacteria (LAB) in inoculated samples. In particular, oleuropein concentration completely disappeared only from samples inoculated with the two selected strains used as starters. The inoculum with selected LAB positively influenced the fermentation process of green olives, whereas the irrigation regime previously experienced by trees did not alter fermentation.

## Introduction

Table olives are mainly produced in Spain, Turkey, Italy, Syria, and Greece, but the market for table olives is expanding well beyond the Mediterranean area. In 2017/18 the world production of table olives is estimated at 2951500 t confirming the increasing trend reported over the last few years (International Olive Council (IOC), 2017). This increase in production was due to good harvests in countries like Egypt, Turkey, Morocco, Argentina, and Tunisia (International Olive Council (IOC), 2017). Table olives are highly appreciated for their sensory characteristics and nutritional value. They also exert potential beneficial effects on human health since they are rich in antioxidant phenols (1–2% of fresh fruit), which are strong free-radical scavengers (Tataridou and Kotzekidou, 2015). Large differences in sensory and nutritional characteristics depend on the genotype and the processing method (Alagna et al., 2012; Kiai and Hafidi, 2014; Ambra et al., 2017). Italy is particularly rich in cultivars for table production, such as Ascolana Tenera, Cellina di Nardò, Itrana, Maiatica di Ferrandina, Nocellara del Belice, Nocellara Etnea (International Olive Council (IOC), 2000), that are widely appreciated as specialty foods. Many of these cultivars, including Itrana, can be actually considered dual purpose because they also produce excellent olive oils. The Itrana cultivar is the most widespread dual purpose (table and oil) cultivar in Latium (Central Italy). For table consumption it is commonly harvested green or black to undergo a process of natural fermentation (Tofalo et al., 2012). Commercially the cultivar Itrana is known as “Gaeta olive” since Gaeta was the port where these products were shipped from to reach far markets. Other factors affecting fruit characteristics include fruit development, crop load, climatic conditions, and cultural practices (Ryan and Robards, 1998; Gucci et al., 2007; Servili et al., 2007; Tura et al., 2008; Alagna et al., 2012).

In Mediterranean countries olive trees are usually grown under rainfed conditions because of the high resistance of *Olea europaea* L. to drought (Gucci and Fereres, 2012). However, exposure to long periods of water scarcity during the summer limits olive productivity (Gucci et al., 2007; Lavee et al., 2007; Gucci and Fereres, 2012; Torres et al., 2017). Irrigation, still seldom used in olive groves for oil production, is more common in plantations for table olive production, mainly because of the positive effects on fruit size, pulp-to-pit ratio, mesocarp cell size, chlorophyll content, productivity, and consequently, the commercial value of olive fruits (Costagli et al., 2003; Gucci et al., 2007, 2009; Lavee et al., 2007; Caruso et al., 2014; Torres et al., 2017). In addition, soil water availability reduces firmness and sugar content of the fruit and affects the concentrations of polyphenols, and secoiridoids in particular, in the oil (Gomez-Rico et al., 2006; Servili et al., 2007; Caruso et al., 2014). In particular, an abundant water supply during fruit development determines lower phenolic concentrations in the oil (Tovar et al., 2002; Gomez-Rico et al., 2006; Servili et al., 2007), probably because of changes both in the biosynthetic and catabolic pathways in the fruit (Alagna et al., 2012; Cirilli et al., 2017).

Water availability represents the main limiting factor for growth and yield of olive trees in the Mediterranean region. However, most papers focused on the evolution of phenolic compounds in olive oil (Gomez-Rico et al., 2006; Servili et al., 2007; Caruso et al., 2014), while no data are available regarding the changes in phenolic concentrations in cultivars for table production, despite the even more critical role played by irrigation on fruit quality and the effect of irrigation on microbial groups and starter cultures efficiency. For instance, it remains to be clarified whether different water regimes can have an impact on the fermentation process and the activity of starter cultures. The use of selected lactic acid bacteria (LAB) strains, associated or not with yeasts, is important since LAB are the main inducers of brine acidification which inhibits the growth of spoilage microorganisms and pathogens and are, therefore, fundamental for the stability of the final product (Corsetti et al., 2012). Moreover, LAB strains reduce debittering time and improve the sensorial and hygienic quality of the final product (Hurtado et al., 2012).

In this study a comparison between the spontaneous and inoculated fermentation processes of naturally fermented table olives from olive trees grown under different irrigation regimes was carried out. The objective of our study was to determine the effect of different irrigation regimes on the fermentation process of Itrana table olives. Two fermentation methods were compared whereby the process progressed spontaneously or was inoculated. In particular, two *L. pentosus* strains (C8 and C11) showing interesting (resistance to NaCl and oleuropein, short debittering time) table olive technological properties (Tofalo et al., 2014; Patent N0. 0001428559) were used as mixed starter cultures. Fermentations were monitored through the determination of pH and microbiological analyses and phenolic composition for 30 days.

## Materials and Methods

### Plant Material and Fruit Sampling

Fruits were harvested on 22 October 2012 from olive (*Olea europaea* L.) trees subjected to different irrigation treatments established in two commercial olive orchards of cv. Itrana located at Fogliano (2 m a.s.l.) and Rocca Massima (375 m a.s.l.) in the Latina province of Latium, Italy. At the Fogliano orchard 6-year-old olive trees (cv. Itrana), spaced at 4 × 4 m, were used (*n* = 3). Water was supplied once a week or every 2 weeks, from July 1 through September 30, using drip lines (1.6 L h^-1^, pressure compensated drippers spaced at 0.6 m). Each tree received about 375 and 750 L during the irrigation season (**Table 1**). At the Rocca Massima orchard 50-year-old olive trees, spaced at 5.5 × 6 m, were grown under either rainfed conditions (RF) or received two complementary irrigations (100 L/tree) on 28 July and 18 August (*n* = 3). Total precipitations from 1 June through 30 September were 202 mm (7, 16, 13, and 144 mm in June, July, August, and September, respectively) and 255 mm (0, 22, 89, and 166 mm in June, July, August, and September, respectively) at Fogliano and Rocca Massima, respectively.

**Table 1:** Coding of treatments for the different locations, irrigation regimes, and starter type.

Treatment	Site	Irrigation	Water applied (L/tree)	Starter
A	Fogliano	Less	375	LAB
Ac		Less	375	Control
B		More	750	LAB
Bc		More	750	Control
C	Rocca Massima	Rainfed	0	LAB
Cc		Rainfed	0	Control
D		Complementary	200	LAB
Dc		Complementary	200	Control

About 5.5 kg of fruits per tree were harvested by hand from different zones of the canopy and carried in refrigerated boxes to the laboratory. An aliquot of 100 fruits was immediately weighed for fresh weigh determination, then oven-dried at 70°C to constant weight, and the oil content in the mesocarp measured by nuclear magnetic resonance using an Oxford MQC-23 analyzer (Oxford /analytical Instruments Ltd., Oxford, United Kingdom) as reported in Caruso et al. (2013). About 3.5 kg of fruits were used for oil extraction and analytical determinations (Caruso et al., 2014).

### Starter Strains and Preparation of Inocula

The two LAB strains (C8 and C11) used in this study were previously isolated from Itrana olive brine and technologically characterized. They were stored at -80°C in de Man, Rogosa and Sharpe (MRS) broth supplemented with glycerol (20% v/v final concentration). Strains belong to the collection of the Faculty of BioScience and Technology for Food, Agriculture and Environment of University of Teramo.

Before inoculation of the olive brine *L. pentosus* C11 and C8 were subcultured overnight at 30°C in MRS broth containing 4% NaCl (wt/vol) for adaptation to the saline environment. After preincubation C11 and C8 strains were centrifuged, washed in a saline solution, and resuspended in sterile brine (water containing 6% NaCl). Each strain was inoculated into the container of olives at a final cell count of approximately 6 log CFU/mL.

### Olive Brining Procedure

The pilot-scale fermentations were performed in triplicate. Samples of healthy olives were washed, selected for a 10–12 mm caliber and processed according to the Greek-type protocol. Before processing, olives with mechanical or insect damage were discarded. Olives were put in sterile vessels, containing 1.5 kg olives and 1.5 L of brine (6% NaCl) and eventually inoculated. The olives were allowed to ferment at ambient temperature, in presence or absence of starter additions. Fermentations were carried out using fruits obtained from individual irrigation trials and the following codes were used (**Table 1**): A (Fogliano less irrigation + LAB); Ac (Fogliano less irrigation); B (Fogliano more irrigation + LAB); Bc (Fogliano more irrigation); C (Rocca Massima rainfed + LAB); Cc (Rocca Massima rainfed); D (Rocca Massima complementary irrigation + LAB); Dc (Rocca Massima complementary irrigation). Fermentations were monitored through the determination of pH and microbiological analyses at different times (0, 7, 15, and 30 days). Phenolic compounds in the olive mesocarp were determined before the beginning and at the end of the fermentation process.

Olive fermentation progress was considered ended when oleuropein disappeared from inoculated samples.

### Microbiological Analysis

Microbiological analyses were performed on brines at different times. Aliquots of 25 mL of brine were diluted with sterile peptone water (0.1% w/v), homogenized with a Stomacher Lab-Blender 400 (Seward Medical, London, United Kingdom) for 2 min, serially diluted and plated in triplicate for microbial enumeration of the following microorganisms: total aerobic mesophilic bacteria (AMB) on Plate count agar (PCA) at 30°C for 2 days; LAB on MRS agar, at 30°C 2 days in microaerophilic conditions and yeasts on Yeast Peptone Dextrose Agar [YPD; 1% (wt/v) yeast extract, 2% (wt/v) peptone, 2% (wt/v) glucose and 2% (wt/v) agar] supplemented with chloramphenicol (150 mg/L) at 25°C for 3 days. The presence/absence of *Salmonella* spp., *Listeria monocytogenes* and *Escherichia coli* O157:H7 was determined according to standard methods (Association Française de Normalisation, 1997; International Organization for Standardization [ISO], 1998, 2002). All media and supplements were provided by Oxoid (Milan, Italy). The analyses were performed in triplicate.

### Physico-Chemical Analysis of Olive Fruits and Brines

pH measurement was carried out on sample (10 mL) of brine using a pH meter MP 220 (Mettler, Toledo, Spain).

Phenolic compounds both olive and brine matrixes were extracted and evaluated by HPLC according to Servili et al. (2008). The concentration was expressed as mg of phenols/Kg of fruits and mg of phenols/L for brines. The dialdehydic form of elenolic acid linked to hydroxytyrosol (3,4-DHPEA-EDA), (+)-1-acetoxypinoresinol and (+)-pinoresinol were isolated from EVOO by semi-preparative HPLC according to Antonini et al. (2015); demethyloleuropein were isolated from the phenolic extract of olive fruit by semi-preparative HPLC according to Servili et al. (1999). Tyrosol (*p*-HPEA) was purchased from Fluka (Milan, Italy), hydroxytyrosol (3,4-DHPEA) was obtained from Cabru s.a.s. (Arcore, Milan, Italy), while the oleuropein and verbascoside were purchased from Extrasynthese (Genay, France). For each compounds were constructed calibration curves to obtain the real concentration.

### Statistical Analysis

One-way analysis of variance followed by the Tukey test were performed by SigmaPlot software package, version 12.3 (Systat Software Inc., San Jose, CA, United States). Windows). Principal component analysis (PCA) was performed using the software XLStat 2014 (Addinsoft, New York, NY, United States).

## Results and Discussion

### Effects of Irrigation on Fruit Characteristics

The use of irrigation was effective in determining changes in fruit characteristics from both sites. At the Fogliano location supplying more water resulted in higher fruit weight (both fresh and dry) (**Table 2**). Both the mesocarp and the endocarp were more developed in fruits from more irrigated trees, but the increase in mesocarp was more than proportional to that of the endocarp and, hence, the mesocarp-to-endocarp ratio of fruits sampled from more irrigated trees was greater. Similar results were obtained at the Rocca Massima site (**Table 2**) and confirmed the importance of irrigation to produce fruit of large size with a high pulp-to-pit ratio (Gucci et al., 2007, 2009; Lavee et al., 2007; Caruso et al., 2013). The different weights in fruits and fruit tissues harvested at the two orchards can be explained by the greater volumes of water received by all trees and the lower crop load at the Fogliano orchard. Interestingly, the mesocarp oil content of the more irrigated trees was significantly greater at both locations (**Table 2**). The oil content is relatively insensitive to soil water availability when the degree of water deficit experienced during fruit development is low, whereas it decreases if stress becomes severe (Gucci et al., 2007).

**Table 2:** Fruit parameters obtained from the different irrigation trials at two locations in 2012.

Fruit parameter	Fogliano		Rocca Massima	
	A	B	C	D
Mesocarp FW (g)	4.78 ± 0.08^a^	3.24 ± 0.09^b^	2.14 ± 0.23^a^	1.30 ± 0.04^b^
Endocarp FW (g)	0.98 ± 0.017^a^	0.73 ± 0.001^b^	0.65 ± 0.06	0.57 ± 0.02
Mesocarp DW (g)	1.16 ± 0.061^a^	0.53 ± 0.004^b^	0.64 ± 0.07^a^	0.40 ± 0.03^b^
Endocarp DW (g)	0.69 ± 0.011^b^	0.82 ± 0.005^a^	0.48 ± 0.03^a^	0.42 ± 0.01^b^
Fruit FW (g)	5.76 ± 0.09^a^	3.97 ± 0.09^b^	2.80 ± 0.30^a^	1.87 ± 0.03^b^
Fruit DW (g)	1.85 ± 0.058^a^	1.35 ± 0.004^b^	1.12 ± 0.10^a^	0.81 ± 0.02^b^
Mesocarp/Endocarp (FW)	4.87 ± 0.10^a^	4.48 ± 0.12^b^	3.30 ± 0.06^a^	2.29 ± 0.14^b^
Fruit DW/Fruit FW	0.32 ± 0.01^b^	0.34 ± 0.01^a^	0.44 ± 0.01^a^	0.4 ± 0.01^b^
Oil in mesocarp (% DW)	60.3 ± 1.54^a^	56.2 ± 1.24^b^	59.3 ± 0.78^a^	51.6 ± 2.35^b^

*Values are means ± standard deviations of three trees for each irrigation treatment. Different letters indicate significant differences (*p* < 0.05) after analysis of variance within each location*.

### Acidification and Microbiological Analyses

**FIGURE 1:**
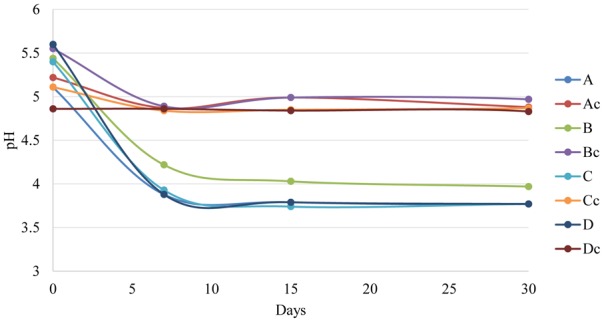
pH evolution during table olive fermentation in inoculated and control samples. A (Fogliano less irrigation + LAB); Ac (Fogliano less irrigation); B (Fogliano more irrigation + LAB); Bc (Fogliano more irrigation); C (Rocca Massima rainfed + LAB); Cc (Rocca Massima rainfed); D (Rocca Massima complementary irrigation + LAB); Dc (Rocca Massima complementary irrigation).

All brine fermentations started with a mean pH value of 5. Within the 7th as apex day of fermentation the pH significantly decreased reaching values of about 4 in all inoculated samples regardless of the irrigation regime. At the end of fermentation, there were no differences among samples with pH values of about 3.7. This finding was consistent with other published data obtained using LAB during Greek- or Spanish-style processing (e.g., *L. plantarum, L. pentosus, L. paracasei*, and *L. rhamnosus*) (De Bellis et al., 2010; Aponte et al., 2012; Blana et al., 2014; Randazzo et al., 2014; Benincasa et al., 2015; De Angelis et al., 2015; Martorana et al., 2017). pH values below 4.5 inhibit the growth of Proteobacteria and other acid-sensitive bacteria avoiding olive spoilage and the development of pathogens during fermentation/storage (Perricone et al., 2010; De Angelis et al., 2015). The pH of uninoculated samples decreased more slowly as expected. After 30 days, control samples reached a value of 4.8 independently from the irrigation regime (**Figure 1**).

**FIGURE 2:**
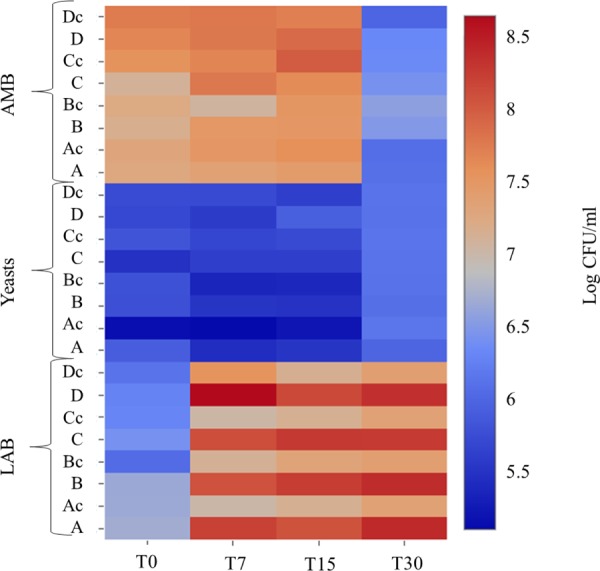
Heatmap showing the distribution of some microbial groups (total aerobic mesophilic bacteria – AMB, LAB, yeasts) in inoculated and control samples during the fermentation process. After an adaptation step C11 and C8 strains were inoculated at a final cell count of approximately 6 log CFU/mL. *Salmonella* spp., *Listeria monocytogenes* and *Escherichia coli* O157:H7 were absent in all samples.

Microbial counts were not affected by the water regime, but showed a shift during fermentation (**Figure 2**). Pathogens were absent in all samples. The initial number of total AMB was about 7 log CFU/mL and then increased during the first 15 days of fermentation, while a decrease after 30 days was observed (about 6 log CFU/mL). This reduction could be related to the low pH values which probably determined a reduction/disappearance of *Enterobacteriaceae* (Panagou et al., 2003; Abriouel et al., 2011). Yeasts populations showed an initial level of about 5 log CFU/mL and increased of about 1 log at the end of fermentation in all samples. Regarding LAB, the initial cell density was about 6 log CFU/mL at T0. In inoculated samples an increase of LAB counts was observed. The water regime did not influence their kinetics since in all inoculated samples the final counts were around 8 log CFU/mL, while in control fermentation vats a final value of 7 log CFU/mL was reached. The more rapid growth of LAB in inoculated samples and their higher count is in agreement with the better acidification rate of these samples compared to control ones. The highest concentration of LAB can be considered a guarantee for the quality of the final product (Corsetti et al., 2012). In fact, the monitoring of pH clearly shows the positive effect of the starter strains. Therefore, we can conclude that the irrigation regime did not influence the fermentation performance of starter strains and, in general, no differences in acidification dynamics and cultivable microbiota growth were evident between the samples obtained under different irrigation treatments.

### Phenolic Compounds Evolution

The phenolic composition is influenced by many factors, such as cultivar, fruit development, climate conditions, and cultural practices including water regime (Servili et al., 2007; Alagna et al., 2012; Caruso et al., 2017; Cirilli et al., 2017). The majority of studies focused on the impact of irrigation system on phenolic compound profile of olive oil, while very few data are available about the impact of water regimes on table olives polyphenolic profiles.

The different irrigation regimes significantly affected the phenolic concentrations in the mesocarp (**Table 3**). Oleuropein was the most abundant phenolic compound and accounted for 48 and 63–68% of total phenolic fractions at the Fogliano and Rocca Massina trials, respectively. Significant differences were observed in the concentrations of the oleuropein, derivative of oleuropein (3,4-DHPEA-EDA) and verbascoside, whereas the lignans (+)-1-acetoxypinoresinol and (+)-pinoresinol were unaffected by the water supply. The irrigation treatments produced an average decrease of the verbascoside content of 36.2%, oleuropein of 9.0%, and 3,4-DHPEA-EDA of 6.3%. Results partially matched those obtained by other authors (Patumi et al., 2002; Tovar et al., 2002; Servili et al., 2007). It is generally accepted that phenolic compounds are more abundant in drought-stressed olive trees than in irrigated ones. These results confirm for cv. Itrana the typical response of secoiridoids concentrations to soil water availability already reported for several other cultivars: as the degree of water deficit becomes more severe phenolic concentrations increase in the fruit and the oil (Tovar et al., 2002; Servili et al., 2007; Caruso et al., 2014). In Greek cultivars, an increase in total phenol content, mainly due to a rise in oleuropein content has been observed under severe water stress (Petridis et al., 2012). A positive relationship between total phenol content and antioxidant activity has also been detected, suggesting that phenols could play a relevant role in the protection against the effects of drought (Petridis et al., 2012). This evidence could be related to drought-related variation in the enzymatic activity of phenylalanine ammonia-lyase (PAL), a key enzyme in the biosynthetic pathway of phenolic compounds, which is directly involved in the accumulation of polyphenols and o-diphenol contents in the olive fruit. It has been reported that the activity of the enzyme PAL in olive fruit decreased with increased irrigation (Patumi et al., 1999; Tovar et al., 2002). Modification of enzymatic activities could explain also other differences. The lower concentration of p-HPEA in drought-stressed samples may be a consequence of decreased activity of the endogenous esterase in the olive fruit that hydrolyzes the bond between p-HPEA and the elenolic acid of ligustroside (Servili et al., 2007).

**Table 3:** Phenolic concentration (mg/kg) of olive fruits of cv. Itrana obtained under different irrigation regimes.

Compound	Fogliano		Rocca Massima	
	A	B	C	D
3,4-DHPEA	495 ± 6.6^a^	479 ± 5.2^b^	175 ± 7.8^a^	400 ± 5.3^b^
p-HPEA	75 ± 5.1	68 ± 2.9	251 ± 21.2^a^	129 ± 8.5^b^
Demethyloleuropein	120 ± 10^a^	220 ± 19.4^b^	352 ± 16.2	345 ± 11.4
Verbascoside	1491 ± 53.5^a^	2498 ± 70.5^b^	908 ± 47.3^a^	1338 ± 69.5^b^
3,4-DHPEA-EDA	3088 ± 28.8^a^	3221 ± 75.7^b^	7300 ± 174.4^a^	8048 ± 231.4^b^
Oleuropein	10354 ± 128.9^a^	11177 ± 177.7^b^	8404 ± 210.1^a^	9408 ± 125.5^b^
(+)-1-acetoxypinoresinol	6 ± 0.1	6 ± 0.4	6.5 ± 0.7	6 ± 0.2
(+)-pinoresinol	1.3 ± 0.1^a^	0.34 ± 0.01^b^	2.2 ± 0.2	2.1 ± 0.1
Σ Phenolic fractions	15633 ± 136.9^a^	17670 ± 205.8^b^	17469 ± 278.5^a^	19677 ± 272.7^b^

*Fruits were harvested by hand on 22 October 2012. Values are means ± standard deviations of three trees for each irrigation treatment. Different letters indicate significant differences (*p* < 0.05) after analysis of variance within each location*.

The debitterig activity of LAB strains (C8 and C11) resulted in a strong decrease of oleuropein, demethyloleuropein, and 3,4-DHPEA-EDA in the olive pulp (**Table 4**). The reduction of oleuropein and 3,4-DHPEA-EDA of inoculated olives was also accompanied by an increase in their hydrolysis products (hydroxytyrosol), confirming the enzymatic activity of these strains affect the secoiridoid glucosides and their aglycon derivatives (3,4-DHPEA-EDA) (Servili et al., 2006, 2008). On the contrary, enzymatic activity did not influence the decrease of verbascoside, indeed in agreement with results reported previously (Servili et al., 2006, 2008) no hydrolytic products (caffeic acid) were found in the olive fruits or brine (data not shown). The reduction of the verbascoside observed in the olives after fermentation, seems to be due to its release in the brine after the process (**Table 5**). The results obtained showed that the microbial combination of the two bacterial strains used was able to carry out the debittering process starting from olives characterized by a different phenolic composition and water regimes. In the current study we also found that the concentrations of total phenols and ortho-diphenols in the oils of more irrigated trees were lower than in rainfed or less irrigated treatments (Supplementary Table S1).

**Table 4:** Phenolic composition (mg/kg) of table olives after 30 days of fermentation.

Compounds	Ac	A	Bc	B	Cc	C	Dc	D
3,4-DHPEA^a^	521.2 ± 33.9^a^	1018.8 ± 55.7^b^	538.3 ± 35^a^	1110.6 ± 60.7^b^	559 ± 37.8^a^	1149.7 ± 92^bc^	388.9 ± 24.2^a^	1296 ± 103.7^c^
*p*-HPEA	58.3 ± 3.8^a^	39.5 ± 1.4^bd^	51.2 ± 3.3^ab^	56.6 ± 4.8^a^	112 ± 7.9^c^	25.1 ± 2.5^d^	206.7 ± 10.9^e^	49.3 ± 2.4^ab^
demethyloleuropein	200.3 ± 11^a^	n.d.	98.2 ± 5.4^b^	n.d.	256.0 ± 10.3^c^	n.d.	226.3 ± 17.1^ac^	n.d.
Verbascoside	2128.9 ± 138.4^a^	1549.4 ± 71.4^b^	1187.1 ± 87.2^c^	1060.3 ± 63.1^ce^	965.7 ± 50.1^e^	846.9 ± 67.8^e^	610.4 ± 31.4^d^	463.2 ± 61.1^d^
3,4-DHPEA-EDA	2788.2 ± 181.2^a^	1323.8 ± 101.4^b^	2682.5 ± 174.4^a^	1216 ± 55.2^b^	6950.9 ± 401.3^c^	3225.1 ± 209.3^a^	6165.1 ± 402.1^d^	1496.5 ± 99.8^b^
Oleuropein	8979.6 ± 493.9^a^	5 ± 0.4^b^	8899.8 ± 489.5^a^	3.5 ± 0.3^b^	8721.9 ± 547.4^a^	n.d.	7502.2 ± 525.2^c^	n.d.
(+)-1-acetoxypinoresinol	4.1 ± 0.1^a^	5.0 ± 0.3^abc^	5.2 ± 0.4^bc^	4.8 ± 0.2^ab^	5.1 ± 0.2^bc^	5.4 ± 0.3^bc^	5.8 ± 0.6^c^	5.3 ± 0.3^bc^
(+)-pinoresinol	1.0 ± 0.1^a^	1.1 ± 0.1^a^	1.8 ± 0.1^bde^	1.7 ± 0.1^bd^	1.9 ± 0.1^bce^	1.5 ± 0.1^d^	2.1 ± 0.2^ce^	2.0 ± 0.1^be^
sum of phenolic fractions	14682 ± 545.2^ae^	3942.6 ± 136^b^	13464.1 ± 528.1^a^	3453.5 ± 103.6^b^	17572.5 ± 681.8^c^	5253.7 ± 238.5^d^	15107.5 ± 662.^9e^	3312.3 ± 156.4^b^

^a^*Data are the mean values of three independent evaluations. Values in each row followed by different letters (a–e) are significantly different from one another at *p* < 0.05. n.d. not detected*.

**Table 5:** Phenolic composition (mg/L) of brines after 30 days of fermentation.

Compounds	Ac	A	Bc	B	Cc	C	Dc	D
3,4-DHPEA^a^	41.8 ± 2.7^a^	648.8 ± 29.7^b^	42.9 ± 2.8^a^	653.2 ± 29.8^b^	154 ± 9.2^c^	1252.3 ± 93^c^	523.6 ± 31.4^d^	916.7 ± 66.9^e^
*p*-HPEA	8.1 ± 0.4^a^	22.1 ± 1.3^b^	24.3 ± 1.2^b^	13.2 ± 0.7^a^	30 ± 1.2^b^	110.8 ± 6.5^c^	7.7 ± 0.3^a^	75.2 ± 5.3^d^
demethyloleuropein	n.d	n.d	n.d	n.d	n.d	n.d	n.d	n.d
Verbascoside	198.7 ± 14.9^a^	614.7 ± 39.2^b^	144.4 ± 9.4^c^	390.8 ± 5.9^d^	256.1 ± 17.4^e^	111.1 ± 8.9^c^	317.6 ± 21.6^f^	118.8 ± 6.5^c^
3,4-DHPEA-EDA	323 ± 21^a^	525.7 ± 27.9^b^	384.9 ± 20.2^a^	530.8 ± 30.2^b^	125 ± 6.3^c^	1043.3 ± 93.1^d^	293.7 ± 14.7^a^	1315.7 ± 65.8^e^
Oleuropein	1117.4 ± 66.5^a^	144.7 ± 10.2^b^	953.7 ± 55.5^c^	158.9 ± 6.9^b^	686.2 ± 44.6^d^	50.4 ± 6.9^e^	419.5 ± 31.8^f^	31.6 ± 2.3^e^
(+)-1-acetoxypinoresinol	n.d	n.d	n.d	n.d	n.d	n.d	n.d	n.d
(+)-pinoresinol	n.d	n.d	n.d	n.d	n.d	n.d	n.d	n.d
sum of phenolic fractions	1689.0 ± 71.4^a^	1956.0 ± 57.5^b^	1550.2 ± 59.9^a^	1746.9 ± 43.4^ab^	1251.3 ± 49.2^c^	2567.9 ± 132.2^d^	1562.1 ± 51.8^a^	2458.0 ± 94.2^d^

^a^*Data are the mean values of three independent evaluations. Values in each row followed by different letters (a–f) are significantly different from one another at *p* < 0.05. n.d. not detected*.

### Statistical Analysis

**FIGURE 3:**
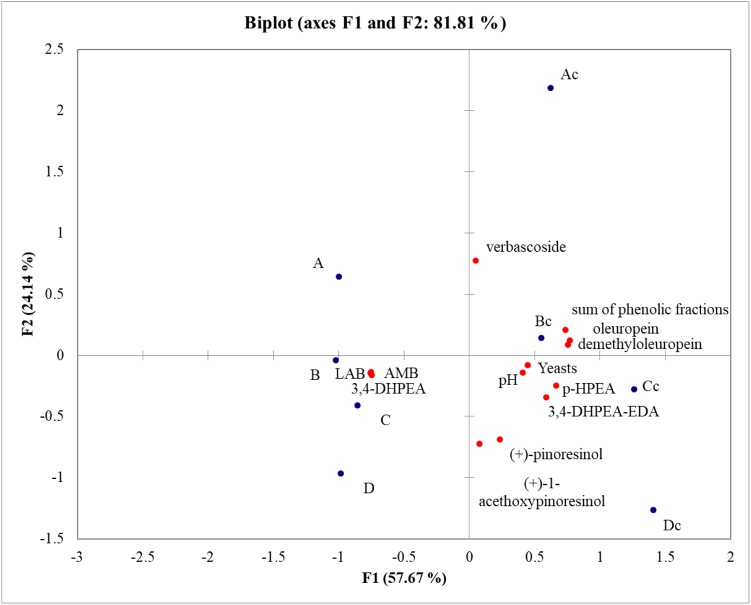
PCA analysis based on brine acidification, microbial groups and amount of phenolic compounds in olives at the end of fermentation.

In order to understand the variability among samples PCA analysis was performed. PCA explained 81.81% of the total variance. F1 accounted for 57.67% of the variance, while F2 explained 24.14% of the variance. Samples were well differentiated and the main differences were observed between inoculated and not inoculated samples. Table olives obtained with the addition of starter cultures were characterized by a high concentration of LAB, AMB, and 3,4 DHPEA. Irrigation system did not influence the fermentation outcome and the main microbial groups, but water availability impacted the amount of phenolic compounds. In fact, Rocca Massima rainfed (Cc) and Rocca Massima complementary irrigation (Dc) samples clustered together and were characterized by a higher concentration of almost all phenolic compounds detected (**Figure 3**).

## Conclusion

This study highlighted the effect of water availability on phenolic compounds profile and fermentation outcome in inoculated and spontaneous fermentation. Microbial dynamics and brine acidification were not influenced by irrigation but only by the inoculation of starter cultures. Selected starter cultures were able to complete the fermentation in 30 days regardless of the irrigation regime, suggesting their adaptation to this ecological niche. Irrigation affected the content of phenolic compounds, which were present in higher concentration in fruits and oils from less irrigated trees. It is important to develop tailored starter culture and optimize irrigation strategies considering some factors such as rainfall seasonality, soil water-holding capacity, and crop evapotranspiration which could influence table olives quality.

## Author Contributions

AlC and RT contributed to the conception and design of the work and supervised all activities. MaS and GP performed microbiological analysis, elaboration, and interpretation of data. AuC, NG-G, and RP prepared starter cultures. RG and GC managed the irrigation plan, fruit sampling and determinations, MuS, SE, and SU evaluated the phenolic content. AlC, RT, MuS, GC, and RG drafted the manuscript. All authors approved the final version of the manuscript to be submitted for publication and agreed to be accountable for all aspects of the work in ensuring that questions related to the accuracy and integrity of any part of the work are appropriately investigated and resolved.

## Conflict of Interest Statement

The authors declare that the research was conducted in the absence of any commercial or financial relationships that could be construed as a potential conflict of interest.

## Abbreviations

LAB: lactic acid bacteria
MRS: de Man, Rogosa and Sharpe
PAL: phenylalanine ammonia-lyase
3,4-DHPEA-EDA: 2-(3,4-hydroxyphenyl)ethyl (3S,4E)-4-formyl-3-(2-oxoethyl)hex-4-enoate.
