# Corrigendum: Changes in Polyphenolic Concentrations of Table Olives (cv. Itrana) Produced Under Different Irrigation Regimes During Spontaneous or Inoculated Fermentation

**DOI:** 10.3389/fmicb.2020.00071

**Published:** 2020-01-31

**Authors:** Giorgia Perpetuini, Giovanni Caruso, Stefania Urbani, Maria Schirone, Sonia Esposto, Aurora Ciarrocchi, Roberta Prete, Natalia Garcia-Gonzalez, Noemi Battistelli, Riccardo Gucci, Maurizio Servili, Rosanna Tofalo, Aldo Corsetti

**Affiliations:** ^1^Faculty of Bioscience and Technology for Food, Agriculture and Environment, University of Teramo, Teramo, Italy; ^2^Department of Agriculture Food and Environment, University of Pisa, Pisa, Italy; ^3^Department of Agricultural Food and Environmental Sciences-DSA3, University of Perugia, Perugia, Italy

**Keywords:** table olive, irrigation, starter cultures, phenolic compounds, oleuropein, Itrana

In the original article, we neglected to include the funder “European Union's Horizon 2020 research and innovation program under the Marie Skłodowska-Curie COFUND, Grant agreement no 713714 to NG-G.”

The authors apologize for this error and state that this does not change the scientific conclusions of the article in any way. The original article has been updated.

