# Supplementary material for: Changes in Polyphenolic Concentrations of Table Olives (cv. Itrana) Produced Under Different Irrigation Regimes During Spontaneous or Inoculated Fermentation
Source: Front Microbiol. 2018 Jun 15;9:1287. doi: 10.3389/fmicb.2018.01287 (PMC6013719; doi:10.3389/fmicb.2018.01287)
Supplement: Supplementary file 1 [file Table_1.pdf]

Table S1. Free acidity, peroxide value, K_{232}, K_{270} ΔK, total bio-phenols and ortho-diphenols in oils obtained from olive trees (cv. Itrana) grown under different irrigation regimes. Values are means ± standard deviations of three replicate trees per treatment. Different letters indicate least significant differences between irrigation treatments after analysis of variance (ANOVA) within each location ($p \leq 0.05$).

**Code**	**Irrigation**	**Free acidity (%. Oleic acid)**	**Peroxide value (meq O_2 kg^{-1})**	**K_{232}**	**K_{270}**	**ΔK**	**Total phenols (mg/kg)**	**Ortho-diphenols (mg/kg)**
A	Less	0.3±0.11	7.3±1.88	1.7±0.08	0.2±0.01^{a}	-0.001±0.0004	338±8.9^{a}	152±5.7^{a}
B	More	0.4±0.17	8.8±1.51	1.8 ±0.06	0.1±0.02^{b}	-0.001±0.0004	311±5.7^{b}	134±6.1^{b}
C	Rainfed	0.3±0.10	5.7±0.92	1.4±0.04	0.1±0.004	-0.005±0.004	220±16.0^{a}	89±7.2
D	Complementary	0.2±0.03	5.9±1.28	1.4±0.10	0.1±0.010	-0.002±0.001	156±22.3^{b}	69±13.0
